# Fatigue symptoms in relation to neuroticism, anxiety-depression, and musculoskeletal pain. A longitudinal twin study

**DOI:** 10.1371/journal.pone.0198594

**Published:** 2018-06-07

**Authors:** Olav Vassend, Espen Røysamb, Christopher Sivert Nielsen, Nikolai Olavi Czajkowski

**Affiliations:** 1 Department of Psychology, University of Oslo, Oslo, Norway; 2 Norwegian Institute of Public Health, Oslo, Norway; 3 Department of Pain Management and Research, Oslo University Hospital, Oslo, Norway; Universitat Wien, AUSTRIA

## Abstract

**Background:**

The nature of the relationship between fatigue and its risk factors is poorly understood. In the present study the genetic and environmental association between anxiety-depression, musculoskeletal (MS) pain and fatigue was examined, and the role of neuroticism as a shared risk factor that may possibly explain the co-occurrence between these phenotypes was investigated in a combined cross-sectional and longitudinal twin design.

**Methods:**

The sample consisted of 746 monozygotic (MZ) and 770 dizygotic (DZ) twins in the age group of 50–65 (mean = 57.11 years, SD = 4.5). Continuous measures of fatigue symptoms and the other phenotypes were employed. Using Cholesky modeling, genetic and environmental influences on the phenotypes, and the associations among them, were determined. Analyses were performed using measures of neuroticism obtained concurrently and 13–19 years earlier.

**Results:**

Results from multiple regression analyses showed that neuroticism, anxiety-depression symptoms, and MS pain were all significantly associated with fatigue, controlling for sex, education, and general health indices. The best-fitting biometric models included additive genetic and individual-specific environmental effects. Heritabilities in the 0.40–0.53 range were demonstrated. Furthermore, while there was a considerable overlap in genetic risk factors between the four phenotypes, a substantial proportion of the genetic risk shared between anxiety-depression and fatigue, and between MS pain and fatigue, was independent of neuroticism.

**Conclusion:**

Evidence for a common underlying susceptibility to report fatigue symptoms, genetically linked to neuroticism, anxiety-depression, and MS pain, was found. Both unique and pleiotropic effects appear to be involved in the genetic architecture of the phenotypes.

## Introduction

Fatigue is a common complaint in clinical settings as well as in the general population [1, 2]. Due to its subjective nature, fatigue is difficult to conceptualize and define, but there seems to be a general agreement that the condition involves a strong and persistent form of mental and/or physical tiredness, weakness, exhaustion, and inability to concentrate [3, 4]. The diagnostic entity chronic fatigue syndrome (CFS) is defined as the presence of impairing fatigue for six months or more, associated with pain and other secondary symptoms and for which no medical or psychiatric causes can be found [4]. While primary care and community studies usually find that between 20% and 40% of the respondents report recent problems with fatigue, CFS conditions are relatively rare [2]. Available evidence suggests that adult community prevalence rates of CFS vary from 0.5% to 4% [5], depending on the assessment methods and the case criteria used. Similar prevalence estimates of prolonged fatigue in community samples of adolescents have been reported [1]. However, the boundary between common fatigue (‘feeling tired and weak’) and diagnosable CFS is quite arbitrary, and many researchers have maintained, also in earlier epidemiological reviews [6], that fatigue is probably best conceptualized as a continuously distributed symptom cluster in the general population [2].

Although several common medical conditions are known to cause fatigue symptoms (e.g., infections, autoimmune disorders, thyroid deficiency, and sleep apnea), a substantial proportion of acute or chronic fatigue conditions remains unexplained [1]. However, several risk factors have been shown to be associated with symptom onset and development: (a) Demographic, lifestyle and environmental factors, including psychosocial and physical working conditions [2]; (b) anxiety-depression symptoms and functional somatic conditions (e.g., irritable bowel syndrome) [7]; (c) personality traits, in particular neuroticism [8]; (d) persistent pain conditions, including both regional and chronic widespread pain (CWP) [9]; and (d) genetic factors [10]., Of note, in fatigue research little attention has generally been paid to the fact that many of the risk factors listed above are inter-related at both the phenotypic and the genetic level [8, 11]. Thus, genetically informative studies are needed that simultaneously examine essential risk factors, such as pain and anxiety-depression symptoms, to determine how they are related to fatigue symptoms.

The relatively few published twin studies of fatigue, mostly of CFS or CFS-like conditions, generally indicate moderate genetic effects, with most heritability estimates ranging from 30% to 50% [12]. Similar heritability estimates have been shown for CFS-like conditions in children [13]. Despite significant sex differences in prevalence of fatigue symptoms or syndromes [5], results from a comprehensive Swedish twin study indicate that the relative importance of genes and environmental factors is fairly similar in males and females [10]. However, a couple of later twin studies suggest non-negligible sex differences in the genetic architecture of fatigue measured both as dichotomous CFS variants [14] and as a continuous symptom measure [15]. Thus, twin research so far provides little consensus with regard to sex differences in genetic and environmental contributors to fatigue.

Epidemiological research, including some longitudinal studies [9, 16], have shown that fatigue is moderately to strongly associated with various psychiatric disorders, in particular anxiety and depression, and general psychological distress. Of significance, chronic fatigue with a co-occurring mood or anxiety state is associated with greater functional impairment than when it occurs alone [1]. Genetic associations between fatigue and depression, anxiety, and general psychological distress were reported by Hickie et al. [12] in what is most likely the first study in this area. Another early study of a small sample of female twins showed that chronic fatigue was strongly associated with psychological distress, but there was no evidence for genetic covariation [17]. Similarly, a recent and much larger study of female twins [9] found no genetic correlation (but a significant individual-specific environmental correlation) between chronic fatigue and major depression. Kato et al. [18] examined underlying genetic and environmental associations between four functional somatic syndromes (CWP, chronic fatigue, irritable bowel syndrome, and recurrent headache) and two psychiatric disorders (major depression and generalized anxiety disorder) in a large twin sample and were able to identify two latent traits in a common pathway model. The first latent trait (characterized by a substantial genetic loading), was common to all six conditions, whereas the other trait (characterized by about equal genetic and individual-specific environmental influences) loaded on all four of the functional somatic syndromes. Thus, the first latent trait indicates that functional somatic syndromes, including fatigue, share underlying mechanisms in part with major depression and generalized anxiety disorder. However, only female twins were included in these biometric analyses. Similar findings were reported in a study of fatigue, insomnia, and depression in another sample of female twins [19]. Finally, a South Asian twin study of three groups of symptoms (psychological, fatigue, and somatic), all measured as continuous variables, demonstrated one latent factor with both genetic and environmental loadings, but with somewhat different parameter values for men and women [15]. Taken together, these studies strongly suggest the existence of genetic associations between fatigue, negative affect symptoms, and somatic complaints or functional somatic disorders. However, the findings are far from unequivocal, and the issue of sex differences in underlying genetic architecture must be characterized as unsettled. It should furthermore be noted that in twin and general epidemiological research in this area, psychometric information (e.g., factor structure and reliability of essential assessment instruments), based on the particular sample investigated, are rarely reported.

Fatigue and pain, and musculoskeletal (MS) pain in particular, often co-occur, and having multiple symptoms may increasingly add to subjective suffering and limitations in functioning [20]. Despite numerous studies of both community and clinical populations, the relationship between pain and fatigue is still poorly understood, and very little genetically informative research on this important public health issue has been published. As mentioned above, Kato et al. [18] demonstrated that the four functional somatic syndromes they studied were genetically related to each other, and to anxiety and depression, but the genetic correlation between CWP and fatigue specifically was not provided. Interestingly, Burri et al. [9] reported strong genetic correlations between CWP on the one hand, and fatigue and depression on the other (the coefficients were 0.78 and 0.63, respectively). As noted, however, their analyses included female twins only, and the authors emphasize that replication studies in independent twin samples are required to further explore the validity and significance of their findings.

The lifespan nature of fatigue conditions and their high prevalence across age groups highlights the need to identify persistent predisposing and perpetuating factors in the pursuit of a multidisciplinary etiological understanding of this health problem. Of particular significance, there is growing evidence that the personality trait of neuroticism—the disposition to experience negative affect such as anxiety, depression, and anger—is a robust correlate and predictor of a wide variety of health-related processes and outcomes [21]. Thus, people who are higher in neuroticism are vulnerable to a broad range of mental and somatic disorders [22], higher levels of comorbidity [23], work-related stress [24], and chronic pain conditions [8]. Moreover, neuroticism seems to influence pain-modulating states such as pain vigilance and catastrophizing [25], and somatic symptom amplification processes in general [26]. Importantly, neuroticism has been shown to be the personality dimension most consistently associated with common fatigue symptoms and CFS or CFS-like illness [16, 27]. Taken together, existing research indicates that neuroticism is best viewed as a general vulnerability factor, which may possibly explain—fully or partly—the association between fatigue and several of its risk factors, including anxiety-depression symptoms and pain.

Although very few twin studies of fatigue and personality have been conducted, their results appear highly consistent. Analyses of longitudinal twin data from the Swedish twin Registry showed that neuroticism, assessed 25 years earlier, predicted chronic fatigue through genetic mechanisms common to the two phenotypes [8, 16]. Similar findings from a cross-sectional twin study were reported by Poeschla and coworkers [28]. To our knowledge, the presence of genetic associations between neuroticism and fatigue symptoms has not been examined in independent, longitudinal replication studies. In one of the studies based on Swedish twin Registry data [8], high levels of neuroticism were also linked to increased risk of developing a variety of somatic conditions, including CWP, ulcers, and headaches. Unfortunately, none of these studies included any of the well-known risk factors for fatigue, particularly anxiety-depression and pain symptoms, in their analyses. Thus, the potentially incremental effect of these risk factors, beyond neuroticism, is unknown.

In the present study we sought to extend the current literature by examining the relationship of fatigue symptoms to neuroticism, anxiety-depression, and MS pain in adult twins aged 50–65. Analyses based on both cross-sectional and longitudinal data, including assessments of neuroticism obtained 13–19 years earlier, were performed. More specifically, the purpose of the study was threefold. First, the prevalence of individual fatigue symptoms as well as the dimensionality (factor number) and reliability of the composite fatigue scale were examined. Second, phenotypic associations were investigated, both zero-order correlations and associations after statistical control for demographic variables and general health indices. Third, biometric twin modelling was employed to determine to what extent common genetic or environmental liability factors contribute to the covariance between the phenotypes. Specifically, the potential role of neuroticism as a shared risk factor that may explain the associations of anxiety-depression and MS pain with fatigue symptoms, was examined.

## Methods

### Participants and data collection

Twins were recruited from the Norwegian Twin Registry (NTR). The registry comprises several cohorts of twins [29], and the current study is based on a random sample from the cohort born 1945–1960. In 2011, questionnaires were sent to a total of 2136 twins. After reminders, 1516 twins responded, yielding a response rate of 71%. Of the participants, 1272 individuals were pair responders, and 244 were single responders. Zygosity has previously been determined based on questionnaire items and has been shown to classify 97–98% of the twins correctly [30]. The cohort, as registered in the NTR, consists only of same-sex twins, and the study sample consisted of 290 monozygotic (MZ) male twins, 247 dizygotic (DZ) male twins, 456 MZ female twins and 523 DZ female twins. Age range of the sample was 50–65 (mean = 57.11, SD = 4.5). This twin cohort also participated in surveys in 1992–98, in which a short form of the Eysenck Personality Questionnaire (EPQ) neuroticism scale [31] was included. Neuroticism was also assessed in 2011, together with anxiety-depression symptoms, MS pain, and fatigue, and the earlier EPQ neuroticism data were added to the 2011 data file. The study was approved by the Regional Committee for Medical and Health Research Ethics—South East Norway, and informed consent was obtained from all participants.

### Measures

As noted, in the 1992–98 surveys neuroticism was assessed using the short form of the EPQ [31, 32]. The EPQ instrument has been employed in several Norwegian studies, including twin studies [33]. The short form of the EPQ neuroticism scale consists of 12 items, and internal consistency reliability (Cronbach’s alpha) in the present sample was 0.80. In 2011, neuroticism was assessed using a Norwegian version of the NEO Personality Inventory Revised (NEO-PI-R) [34, 35]. Internal consistency reliability (Cronbach’s alpha) for the six facets comprising the neuroticism domain was 0.84 in this sample. Accumulated research has shown that personality traits, including neuroticism, are highly stable across the life span, particularly in adulthood [36]. Corrected for attenuation due to measurement errors, stability coefficients in the 0.82–0.94 range have been reported in meta-analyses [37].

Fatigue and MS pain symptoms were measured using the Giessen Symptom Checklist (GSCL) [38, 39]. The GSCL has been widely used in epidemiological research (e.g., [40]), but also in experimental studies [41]. The fatigue sub-scale comprises 6 items: (1) Physical weakness, (2) excessive need for sleep, (3) tendency to rapid exhaustion, (4) tiredness or drowsiness, (5) feeling distant, difficulty concentrating, and (6) feeling of listlessness. The participants were asked to rate the degree to which they ‘generally’ suffered from the complaints, using a 5-point scale: 1—not at all; 2—slightly; 3—somewhat; 4—considerably; and 5—strongly. Internal consistency reliability was very good (Chronbach’s alpha = 0.88).

Pain symptoms were measured using 3 items from the MS sub-scale of the GSCL. These items were (1) pains in joints or limbs, (2) backache, and (3) pain in neck and shoulders. Additional 3 symptoms (i.e., headaches, heaviness or tiredness in the legs, and head-pressure) are included in the original full MS complaints sub-scale of the GSCL [38]. In the present study, however, in which MS pain symptoms in the strict sense constitute one of the main study variables, and content (item) overlap of fatigue and MS symptoms is to be avoided, results based on the shortened scale (i.e., the mean of the 3 first items) will be reported. Internal consistency reliability for this scale was acceptable (Chronbach’s alpha = 0.76).

Depression-anxiety symptoms (in 2011) were assessed using 5 questions from the Symptom Checklist 25 (SCL-25), which is a shortened version of an originally 90-item questionnaire designed by Derogatis and coworkers [42, 43]. The twins were asked if they during the last 14 days had been (1) feeling fearful; (2) feeling tense or keyed up; (3) feeling hopeless about the future; (4) feeling blue; and (5) worrying too much about things. Each item was rated on a 4-point scale: 1 –not at all; 2 –a little bit; 3 –quite a bit; and 4 –extremely. The sum of these 5 items (SCL-5) has been shown to correlate 0.92 with the total SCL-25 score [33]. The SCL-5 has been used in previous Norwegian twin studies of e.g. back-neck pain [44] and internalizing disorders [45]. The latter study [45], which comprised nearly 8000 adult twins, showed a strong genetic correlation of 0.82 between current self-reported (SCL-5) anxiety-depression symptoms, and lifetime internalizing disorders, suggesting a substantial overlap in genetic liability. Internal consistency for the SCL-5 in the present sample was very good (Chronbach’s alpha = 0.87).

The demographic variables included in the study were sex and education (5 levels). Age was not significantly associated with any of the main variables, probably due to restricted age range in this particular cohort, and was consequently not included as a covariate in the analyses. A set of general health indices was adopted from previous large-scale Norwegian health surveys [40, 46]. These were (1) presence of known medical disease (yes/no), (2) presence of lasting functional impairment (yes/no), and (3) reduced activity or days in bed due to illness (acute or chronic) or injury for the last two-week period (yes/no), before completing the questionnaire.

### Statistical analyses

Initially, the distributions of the individual fatigue symptoms and the prevalence of symptomatic individuals were examined. Next, exploratory factor analysis with maximum likelihood extraction was used to determine the dimensionality (number of factors) underlying the item covariance structure. Three common factor selection procedures were employed: (1) the eigenvalues > 1.0 rule, (2) the scree test, and (3) parallel analysis. Due to the non-independence of observations within samples of genetically related individuals, the correlation and factor analyses were carried out in two sub-samples comprising, respectively, twin 1 and twin 2 from the twin pairs. Correlations among the study variables were then inspected, and multiple regression analysis based on the total sample and the twin 1 and twin 2 sub-samples separately were conducted to examine the independent effects of neuroticism, anxiety-depression symptoms and MS pain on the total (mean) fatigue symptoms score in a model also including demographic and general health variables. Because regression analysis needs to reflect the paired structure of the data when the complete sample is examined, Generalized Estimating Equations (GEE) were used [47].

**Fig 1:**
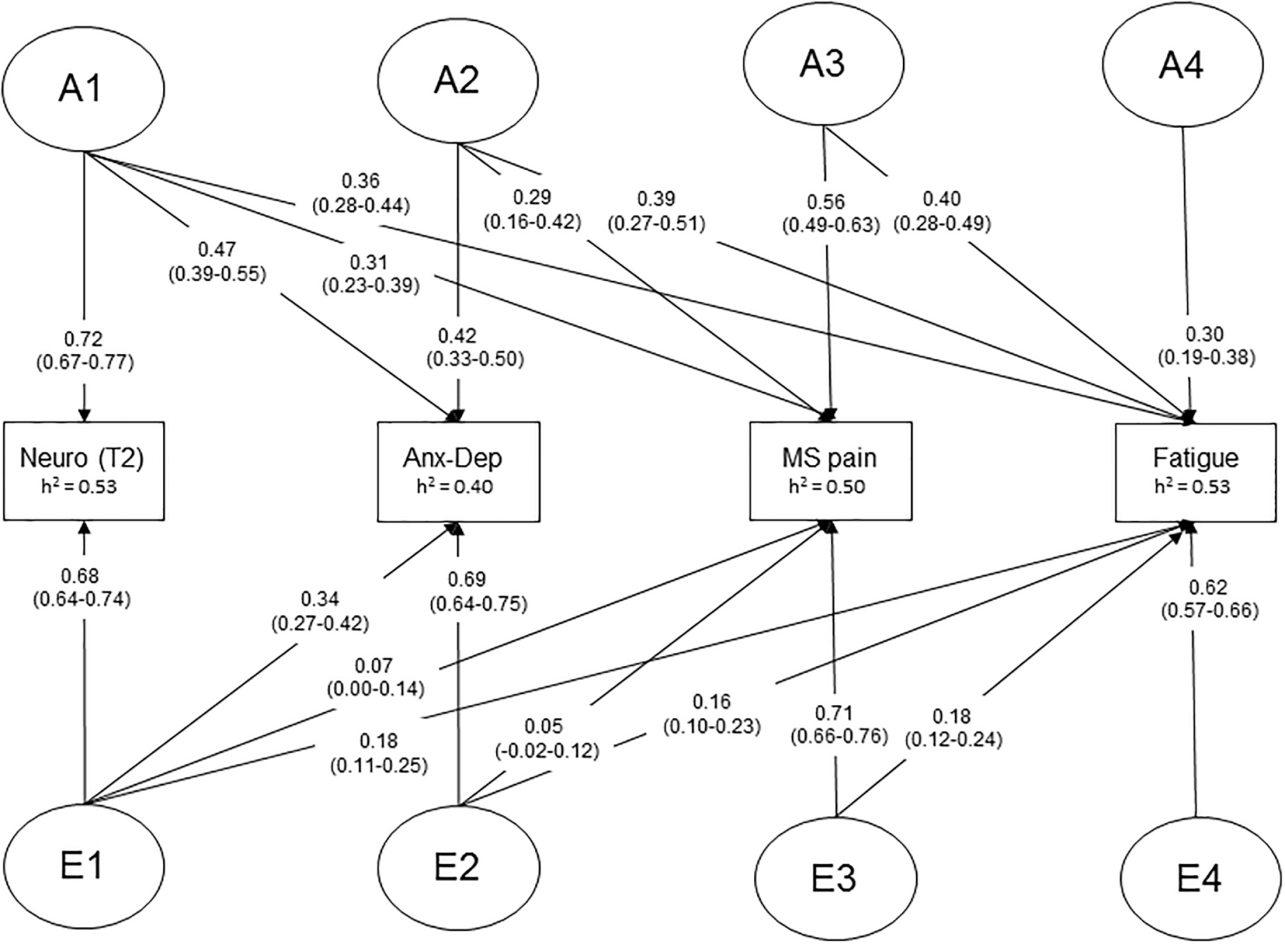
Path diagram of the best-fitting AE Cholesky model. The model is depicting genetic (A) and environmental (E) influences on the phenotypes neuroticism (Neuro, assessed at time 2: 2011), anxiety-depression (Anx-Dep) symptoms, musculoskeletal (MS) pain, and fatigue. 95% CI in parentheses. h^2^ –heritability coefficient.

In the biometric analyses standard Cholesky models [48, 49] were used to estimate the genetic and environmental contributions to variance in and covariance between neuroticism, anxiety-depression symptoms, MS pain, and fatigue. All models were run with OpenMx [50]. Generally, biometric modeling allows for estimating three major sources of variance, i.e., additive genetic factors (A), common environment (C), and non-shared or individual-specific (E) environment. In addition, non-additive genetic effects (D) may be tested, but are only indicated if the observed MZ-correlations are greater than twice the DZ-correlations. Models are constrained so that latent A-factors correlate perfectly among MZ-twins, and at 0.5 among DZ-twins. C-factors are correlated at unity for both zygosity groups, and E-factors are by definition uncorrelated. The Cholesky model specifies as many latent genetic and environmental factors as observed variables (phenotypes) in a triangular decomposition (for illustration see Fig 1). Thus, the genetic factor A1 (Fig 1) influences the neuroticism trait and the three other phenotypes, whereas factor A2 influences anxiety-depression, MS pain, and fatigue, controlling for neuroticism, and likewise for A3 and A4.

Several nested models were compared in order to identify the best-fitting one according to the minus2LogLikelihood difference test (Δ-2LL) and the Akaike Information Criterion (AIC) [51]. Thus, an ACE model was compared to an AE model, and the consequences of constraining the parameters to be equal across sex in a given model were assessed. In investigating potentially sex-limited effects of genetic and environmental factors using the Cholesky model, the approach outlined by Neale et al. [48] was adopted. In same-sex twin samples only so-called common effects sex-limitation can be assessed, i.e., it is presupposed that the same factors cause variation in males and females, but they may do so to a different extent. Hence, the genetic and environmental correlation matrices must be constrained to be equal for males and females. In the scalar model, which is a sub-model of the common effects model, the sex-specific effects are removed and the variance components (A, C, E) for females are all constrained to be equal to a scalar multiple of the male variance components. In consequence, the standardized variance components are equal across sex even if the unstandardized components may differ. In the first two models, parameters were estimated separately for males and females (common effects sex limitation). In models 3 and 4, scalar sex limitation was examined, and in models 5 and 6 all structural parameters were constrained to be equal across sex (no sex limitation). The model with the lowest AIC represents the best balance of goodness-of-fit and parsimony.

### Results

Although the mean fatigue symptom level was rather low (mean = 1.51; SD = 0.42), the percentage of symptomatic individuals (i.e., those reporting above 1 –‘not at all’ on the symptom scales) was considerable, ranging from 27.4% (for listlessness) to 43.9% (for tiredness or drowsiness) (Table 1). Depending on symptom type, between 2.1% and 6.3% of the respondents indicated that they suffered ‘considerably’ or ‘strongly’ from their complaints. Furthermore, nearly 63% of the participants reported two or more symptoms, and about 25% reported four to six (maximum) symptoms (data not shown). Mean symptom level was significantly higher for women compared to men (1.56 and 1.41, respectively, p <0.001), but no significant difference between the MZ and DZ groups was observed (data not shown).

**Table 1:** Score distributions (percentages) for the six symptoms included in the fatigue index, and prevalence of symptomatic individuals (i.e., with a score equal to or greater than 2: Slightly).

	1: Not at all	2: Slightly	3: Somewhat	4: Considerably	5: Strongly	% symptomatic
1. Physical weakness	68.0	18.7	8.5	3.9	0.9	32.0
2. Excessive need for sleep	66.4	19.8	8.4	3.1	2.4	33.6
3. Tendency to rapid exhaustion	65.1	20.1	8.5	4.2	2.1	34.9
4. Tiredness/drowsiness	56.1	29.7	9.3	2.9	2.0	43.9
5. Feeling unconcentrated/distant	72.4	21.0	4.5	1.8	0.3	27.6
6. Listlessness	72.6	20.2	4.7	1.8	0.8	27.4

Factor analysis of the fatigue symptoms yielded only one factor as judged by the eigenvalue >1 criterion, and scree plot inspection (data not shown). Parallel analysis led to the same conclusion, i.e., plotted eigenvalues from random data crossed the plot of eigenvalues from the sample data (the scree plot) between the first and the second factor (data not shown). The first/second eigenvalues in the two sub-samples were 3.75/0.67 and 3.89/0.69, respectively, and the factor loadings were in the 0.60–0.85 range. This common ‘fatigue symptom factor’ explained 62% and 65%, respectively, of the symptom variance in the twin 1 and twin 2 sub-samples, and may thus be characterized as both salient (in terms of explained variance) and well-defined (in terms of factor loadings).

Next, the phenotypic relationship between fatigue symptoms, neuroticism, anxiety-depression, and MS pain was examined in correlation and regression analyses (Tables 2 and 3).

**Table 2:** Phenotypic correlations.

	1. Neuroticism (time 1)	2. Neuroticism (time 2)	3. Anxiety-depression	4. MS pain symptoms
1. Neuroticism (time 1)				
2. Neuroticism (time 2)	0.54 (0.51–0.56)			
3. Anxiety-depression	0.47 (0.44–0.50)	0.58 (0.55–0.60)		
4. MS pain symptoms	0.26 (0.22–0.29)	0.28 (0.25–0.32)	0.32 (0.29–0.36)	
5. Fatigue	0.33 (0.30–0.37)	0.39 (0.36–0.42)	0.50 (0.47–0.53)	0.60 (0.58–0.62)

Time 1—(1992–1998); Time 2—(2011); MS—Musculoskeletal. Pearson correlation coefficients; 95% CI in parentheses.

**Table 3:** Risk factors for fatigue.

1. Variable	2. Mean (SD) % (dichotomous variables)	3. Regression coefficients (Neuroticism time 1)	4. Regression coefficients (Neuroticism time 2)
Neuroticism (time 1/time 2)	1.80 (0.22)/1.58 (0.42)	0.17*	0.12*
Anxiety-depression	1.32 (0.48)	0.40***	0.40***
MS pain symptoms	1.98 (0.92	0.31***	0.32***
Education	3.21 (1.32)	0.00 (ns)	0.01 (ns)
Sex (women, %)	64.6	-0.01(ns)	0.00 (ns)
Reduced activity/in bed (%)	11.3	0.24***	0.22***
Medical condition (%)	40.9	0.14***	0.15***
Functional impairment (%)	8.6	0.23***	0.24***

Descriptive statistics (second column) and estimates of regression coefficients based on generalized estimating equations, representing statistical effects of independent variables on fatigue. Results from analyses including neuroticism scores obtained at time 1 and time 2 are shown in the third and fourth column, respectively (the other independent variables are identical in the two analyses). Time 1—(1992–1998); Time 2—(2011); MS—Musculoskeletal;

* p < 0.05;

*** p < 0.001; ns—not significant.

Descriptive statistics for the study variables and the covariates are shown in Table 3. As expected for this non-clinical sample of twins, anxiety-depression and MS pain symptom levels at the lower end of the scales were found. Yet, a large proportion (i.e., 40.9%) of the participants reported having at least one medical condition. The highest phenotypic correlation was observed between fatigue and MS symptoms (r = 0.60, p < 0.001), and the other phenotypic correlations ranged between 0.28 and 0.54. In addition, female sex and lower education were associated with fatigue (r = -0.11 and r = -0.20, respectively; p < 0.001).

In multiple regression analysis (GEE), both neuroticism (time 1 and time 2), anxiety-depression symptoms, and MS pain were found to be significantly associated with the fatigue index (Table 3). Furthermore, all the general health indices also yielded significant statistical effects on the dependent variable. The associations of sex and education with fatigue symptoms were no longer significant. In hierarchical regression analyses conducted separately in the twin 1 and twin 2 sub-samples, neuroticism assessed at time 2 (NEO-PI-R), anxiety-depression and MS pain symptoms accounted for about 58% of the variance in fatigue symptoms in both sub-samples when entered together in the first block of variables. When the general health indices were included as independent variables in the second block, the amount of explained variance increased to just about 60%. In analyses using neuroticism measured at time 1 (EPQ), the amount of explained variance in the first/second block of variables were 47%/51% and 49%/53%, respectively, in the twin 1 and twin 2 sub-samples. In order to explore to what extent the regression parameter values are influenced by extreme scorers, the GEE analyses were rerun, excluding participants with a mean score of 3 (‘considerable’) or above on the fatigue scale. In these analyses, in which about 5% of the total sample were left out, the regression coefficients for neuroticism, anxiety-depression, and MS pain were still significant, with only a small reduction or no reduction in the parameter values for neuroticism at time 1 and time 2, respectively (data not shown).

In the multivariate biometric analyses, a set of Cholesky models including neuroticism, anxiety-depression, MS pain and fatigue was tested. Table 4 shows the fit of the different models including neuroticism and the other study variables assessed simultaneously (i.e., at time 2). As can be seen, for each pair of ACE-AE models (i.e., the following pairs of the models listed in Table 4: 1 vs. 2; 3 vs. 4; 5 vs. 6), the AE model produced the best fit. Moreover, as compared with the first four models (common effects/scalar sex limitation), model 5 and 6 (no sex limitation) resulted in a significant worsening of fit as judged by both AIC and the Δ-2LL test. Thus, some form of sex limitation appears necessary to achieve an adequate model fit, with model 4 (AE) having lowest AIC and thus designated as the best-fitting model.

**Table 4:** Model fitting results for model including neuroticism (time 2), anxiety-depression symptoms, MS pain and fatigue.

Model	-2 log likelihood	df	Δ-2LL (Δdf)	AIC
1. ACE (sex-specific parameters)	8571.44	5960	-	-3348.56
2. AE (sex-specific parameters)	8583.45	5980	10.00 (20)	-3376.55
3. AEC (scalar sex limitation)	8588.87	5974	17.52 (14)	-3359.03
4. AE (scalar sex limitation)	8589.86	5984	18.41 (24)	-**3378.15**
5. AEC (no sex limitation)	8696.28	5978	124.83 (18)	-3259.72
6. AE (no sex limitation)	8697.35	5988	125.90 (18)	-3278.66

A—Additive genetic effects; C—Common environmental effects; E—Individual-specific environmental effects; AIC—Akaike’s Information Criterion; time 2—2011; MS—Musculoskeletal. Best-fitting model shown in bold.

The Cholesky models were then rerun, now including neuroticism at time 1, in addition to the other study variables. Again, for each pair of ACE-AE models, the AE model produced the best fit (data not shown). However, the two best-fitting models, i.e., model 2 and model 4, demonstrated essentially the same degree of fit (Δ-2LL = 8.63, Δdf = 4, ns; AIC = -4687.86, and AIC = -4687.23, respectively). For comparison purposes, parameter values based on model 4 will be reported. Finally, a set of models was run that included both time 1 and time 2 neuroticism measures simultaneously, in addition to MS pain and fatigue symptoms. In these analyses, model 4 provided the best balance of fit and parsimony of the observed data (AIC = -5138.80), with model 2 emerging as the second-best model (AIC = -5135.73). A strong genetic correlation (r_g_ = 0.79, p < 0.05) between the two neuroticism measures was found, in addition to a much weaker, but significant individual-specific environmental correlation (r_e_ = 0.28, p < 0.05). Probably due to this substantial genetic commonality the genetic cross-effects of neuroticism at time 2 (NEO-PI-R) on MS pain and fatigue were no longer significant (data not shown). However, a small but significant individual-specific environmental effect (i.e., 0.16) of neuroticism (time 2) on fatigue, but not on MS pain, was found.

Fig 1 shows the standardized Cholesky parameters of the scalar sex limitation (AE) model based on simultaneous (time 2) assessments of the four phenotypes (i.e., model 4 in Table 4). A genetic factor (A1) accounted for 53% of the total variance in neuroticism (NEO-PI-R), 22% of the variance in anxiety-depression symptoms, 10% of the variance in MS pain, and 13%, of the variance in fatigue. A second genetic factor (A2), independent of A1, accounted for an additional 18% of the variance in anxiety-depression symptoms, and 8% in MS pain and 15% in fatigue. The third genetic factor (A3) accounted for additional 31% of the variance in MS pain and 16% of the variance in fatigue. Finally, there was also a modest amount of specific genetic variance for fatigue (A4), explaining 9% of its variance. Moderate to strong heritability estimates for the four phenotypes were found (Fig 1). In contrast to the distinct and fairly uniform pattern of genetic associations emerging from these analyses, environmental effects (i.e., the cross-effects of E1, E2 and E3) were generally much smaller and did not form a particularly homogenous pattern. Thus, while moderate cross-effects of E1 on anxiety-depression symptoms were observed (Fig 1), this environmental factor accounted for only a trivial amount of the variance in fatigue symptoms (3%) and a non-significant amount of variance in MS pain. Furthermore, the E2 factor also accounted for just a small amount of the variance in fatigue symptoms (3%), and its influence on MS pain was non-significant. The effect of the E3 factor on fatigue was equal to the effects of the E1 and E2 factors, accounting for 3% of the phenotypic variance. Taken together, however, these results indicate that there are small, but non-negligible individual-specific environmental influences of neuroticism, anxiety depression, and MS pain on fatigue. Finally, the total individual-specific environmental effects on each phenotype were substantial, explaining a large amount of the variance in neuroticism (47%), anxiety-depression symptoms (49%), MS pain (50%), and fatigue symptoms (47%).

Parameter estimates based on the second AE Cholesky model (including neuroticism assessed at time 1) were broadly similar to the ones observed for the first AE model, and particularly the genetic effects (S1 Fig). The cross-effects of the E1 factor in this AE model were even smaller than the corresponding E1 effects in the first AE model, accounting for less than 1% of the variance in fatigue. In contrast, the genetic component of neuroticism, measured 13–19 years earlier or concurrently (time1/time2), accounted for a substantial and comparable amount of the total variance in anxiety-depression (26%/22%), MS pain (10%/10%), and fatigue (15%/13%). The other genetic and environmental effects in the second AE model were almost identical to the corresponding effects in the first AE model.

Genetic and individual-specific environmental correlations generated from the model including neuroticism (time 2), anxiety-depression symptoms, MS and fatigue symptoms are shown in Table 5. As can be seen, the genetic correlations are substantial and considerably larger than the environmental correlations. A strong genetic correlation between anxiety-depression symptoms and neuroticism could be detected, as well as more moderate genetic correlations between neuroticism, and MS pain and fatigue. Furthermore, there were moderate to strong genetic correlations among anxiety-depression symptoms, MS pain and fatigue. Significant individual-specific environmental correlations could be observed between fatigue on the one hand, and neuroticism, anxiety-depression symptoms, and MS pain, on the other. The highest environmental correlation was between neuroticism and anxiety-depression symptoms. The genetic correlations between time 1 neuroticism scores and anxiety-depression symptoms was 0.80 (0.68–0.92), between neuroticism and MS pain 0.43 (0.30–0.57), and between neuroticism and fatigue 0.54 (0.42–0.69). The corresponding environmental correlations were 0.22 (0.12–0.32), 0.06 (-0.03–0.16), and 0.12 (0.01–0.21).

**Table 5:** Genetic correlations (above diagonal) and individual-specific environmental correlations (below diagonal) between the phenotypes.

	1. Neuroticism (time 2)	2. Anxiety-depression	3. MS Pain symptoms	4. Fatigue
1. Neuroticism (time 2)		0.75 (0.65–0.84)	0.45 (0.33–0.56)	0.50 (0.39–0.60
2. Anxiety-depression	0.44 (0.36–0.52)		0.60 (0.47–0.73)	0.73 (0.62–0.83)
3. MS pain symptoms	0.09 (-0.004–0.19)	0.10 (0.003–0.20		0.88 (0.80–0.95)
4. Fatigue	0.26 (0.16–0.35)	0.33 (0.23–0.41)	0.30 (0.21–0.39)	

Time 2—2011; MS—Musculoskeletal; 95% CI in parentheses.

## Discussion

Our study contributes to the knowledge of structure, correlates, and etiology of fatigue symptoms in primarily two ways. First, the relationship of fatigue to neuroticism, anxiety-depression and MS pain was investigated, for the first time we assume, in a genetically informative, combined cross-sectional and longitudinal design. Second, the distributions and psychometric structure of common fatigue symptoms were examined.

### Relation to previous research

In congruence with previous psychometric research on the GSCL instrument [38, 39], a one-factor model was shown to be an appropriate measurement model for the fatigue symptoms sub-scale. Furthermore, on the phenotypic level neuroticism (assessed concurrently or 13–19 years previously), anxiety-depression symptoms and MS pain were significantly and robustly associated with fatigue. Biometric modeling showed that all phenotypes were at least moderately heritable (h^2^ ranging from 40% to 53%), and the best-fitting model comprised only A and E factors. With regard to the fatigue symptom index, the estimated heritability of 0.53 matched earlier reports [12]. Extending previous research, Cholesky modeling revealed significant genetic cross-effects, indicating that shared genetic etiology (pleiotropy) produces associations among the phenotypes, but also that the genetic overlap between anxiety-depression levels and fatigue, and between MS pain and fatigue, is not solely due to their relationships with neuroticism. Importantly, the independent genetic influence of MS pain on fatigue was substantial, and the genetic correlation between the two phenotypes was especially strong (r_g_ = 0.88). Overall, our results concur with previous twin studies in this area, which have examined a subset of the phenotypes included in the present study, but not all four phenotypes simultaneously. Moreover, as shown by Hickie et al. [12], while fatigue and psychological distress share some common genetic factors, fatigue has also substantial independent genetic and environmental risk factors.

A large proportion of the participants (i.e., between 20% and 30%) indicated that they were just ‘slightly’ affected by fatigue symptoms. Of particular note, however, between 2.1% and 6.3% (depending on symptom type) reported that they suffered ‘considerably’ or ‘strongly’ from their symptoms. Such symptom loads are obviously of clinical significance, and the percentage of individuals falling into this category is highly similar to prevalence estimates of CFS or CFS-like illness reported in general population studies [1, 10]. Importantly, however, when excluding individuals with a probable fatigue disorder (i.e., with scores ≥ 3 on the fatigue scale), the associations of fatigue with neuroticism, anxiety-depression, and MS pain remained significant and the changes in regression parameter values were small to moderate. Thus, the phenotypic associations are not caused solely by a relatively small group of extreme scorers, which argues for the use of a continuous phenotype definition.

### The role of sex and general health status

As expected, general health variables were significantly associated with fatigue symptoms in bivariate analyses and when entered simultaneously as independent variables in multiple regression analyses. However, hierarchical regression analyses conducted separately in the two twin sub-samples revealed that these variables accounted for just a tiny proportion of the variance in fatigue symptoms when entered after neuroticism, anxiety-depression, and MS symptoms, which together accounted for more than 50% of the variance. In accordance with previous epidemiological research [1, 2], women evidenced a higher fatigue complaint level than men. However, this sex effect was no longer significant after adjustment for education, general health indices, and the other main study variables. Evidently, a substantial part of the sex difference in fatigue symptoms can be attributed to sex differences in other phenotypic characteristics, in particular neuroticism [8], various pain conditions, including MS pain [52], and anxiety-depression and other negative affect symptoms [18]. Furthermore, while phenotypic variance was generally somewhat larger for women than men, in biometric modeling standardized A and E parameters could be set equal across sex without a resulting reduction in model fit. These results largely confirm findings from at least two other twin studies of fatigue [10, 12], one of which also including anxiety, depression, and general psychological distress [12]. As noted, studies based on very large samples drawn from the Swedish Twin Registry have shown that although the risks for all phenotype definitions of fatigue (ranging from any fatigue to CFS-like illness) were between two to four times higher in females [5], the genetic architecture of these phenotypes was highly similar across the sexes [10].

### Theoretical models

A strongly influential common genetic factor was identified, accounting for about 50% of the total variance in neuroticism and between 10% and 25%, of the variance in negative affect, MS and fatigue symptoms. This factor may reflect a general susceptibility to psychological and somatic distress, which is a core characteristic of the neuroticism trait [23, 26]. Interestingly, similar findings were reported by Hansell and colleagues [53] in a study of adolescent twins. They showed that the relationship between neuroticism, anxiety-depressions symptoms, and perceived somatic health was to a large extent due to a common genetic factor. Importantly, additional genetic covariation between anxiety-depression and somatic health, independent of neuroticism, was demonstrated.

A general explanation for the link between neuroticism and health outcomes is one proposed by Eysenck [31], stating that chronic emotional instability and activation result in psychological distress and physiological ‘wear and tear’. According to the concept of allostasis, a later contribution to stress theory [54], an organism seeks to maximize adaptation and the probability of survival by changing parameters of its internal physiological milieu, matching them to the perceived environmental demands [55]. However, recurrent or chronic allostatic activity (i.e., stress responses) leads to systemic somatic damage, reduced mental health and well-being, and loss of resilience to additional stressors [56]. These accumulating effects are termed allostatic load and are generally measured using multisystem biomarker composites, representing e.g., cardiovascular, immune, metabolic, and neuroendocrine systems. There is currently only scant research available on the relationship of allostatic load with fatigue and neuroticism. In a population-based case-control study, Maloney et al. [57] showed that, compared with healthy controls, persons with CFS or CFS-like conditions were significantly more likely to have a high allostatic load. In a recent longitudinal study, Stephan et al. [58] found that neuroticism was linked to allostatic load at baseline, but higher allostatic load was not associated with increases in neuroticism over the 4-year study period. The present study confirms the pervasive relationship between neuroticism and both mental and physical health indices. Whatever the underlying mechanisms might be, in line with previous twin research [8, 53] our findings indicate that the phenotypic associations are primarily mediated by shared genetic influences.

It is now possible to test for such pleiotropy in associations between fatigue, neuroticism and various health indices using data from single nucleotide polymorphism (SNP) genotyping in genetically unrelated individuals. In a newly published study, probably the first of its kind and using data on more than 100 000 participants in the UK Biobank, Deary and colleagues [59] carried out a genome-wide association study (GWAS) of responses to a single question regarding tiredness/low energy (“Over the last two weeks, how often have you felt tired or had little energy?”). Significant genetic correlations were identified between self-reported tiredness and psychological/psychopathological traits (e.g., major depressive disorder, bipolar disorder, schizophrenia, verbal-numerical reasoning, and neuroticism), as well as various biomedical and health behavior indices (e.g., body mass index [BMI], C-reactive protein, forced expiratory volume, smoking status, and triglycerides). Of note, particularly strong genetic correlations between tiredness, and depressive disorder (r_g_ = 0.59) and neuroticism (r_g_ = 0.62) were demonstrated. The proportion of variance explained by all common SNPs for the tiredness question was 8.4%, and significant associations were identified between tiredness phenotypic scores and polygenic profile scores for several of the physical and psychological traits, including depressive disorder and neuroticism. The authors maintain that tiredness appears to be genetically linked to a systemic proneness to poor health as shown particularly by its genetic associations with markers of allostatic load. They furthermore suggest that neuroticism may represent a separate route to fatigue, a predominantly affective one, but with a probable overlap with biomedical mechanisms and risk factors mentioned above. As evidence for this hypothesis, the authors mention that when polygenic profile score analyses of tiredness were adjusted for neuroticism, the associations between tiredness and mental disorders (except schizophrenia) were largely attenuated, whereas most of the biomedical/behavior health associations remained significant. These results suggest, firstly, that it is the proneness to neuroticism, rather than the specific proneness to mental disorders, that may account for the relationship between tiredness and mental health indices. Secondly, they show that while neuroticism is associated with some of the physical health-related traits included in the UK Biobank [60], the genetic associations between tiredness and biomedical/health behavior traits identified by Deary et al. [59] are evidently not wholly confounded by neuroticism level.

Previous research into the genetics of fatigue has primarily focused on genes associated with biological mechanisms hypothesized to be implicated in the disorder, particularly genes involved in the immune system, the hypothalamic-pituitary-adrenal (HPA) axis, and the serotonergic system. In a review of this literature, Landmark-Høyvik et al. [61] concluded that the search for genetic markers of fatigue has been unproductive, and most studies are hampered by lack of power, phenotypic heterogeneity, and poor study design. In a recent review [62], several potentially important associations of SNPs related to neurotransmitter systems, the HPA axis, and immune-mediated inflammation with fatigue, including CFS and disease-related fatigue, were discussed. Of note, the review suggest a major role for cytokine SNPs in all the fatigue subgroups. The authors point out, however, (1) that sample sizes for all the studies included were relatively small; (2) that many studies lack an accurate case-definition; (3) that there are relatively few longitudinal studies; and (4) that no GWAS studies were found. Regrettably, the Deary et al. [59] study did not include measures of cytokines, HPA axis activity, or pain symptoms. Thus, large-scale studies of genetic markers of various definitions of fatigue, and genetic markers shared with phenotypes and endophenotypes associated with fatigue, are needed to attain a better understanding of the genetic architecture and etiological mechanisms involved in the disorder.

Results from the Cholesky analyses also demonstrated weaker, but significant environmental influences (E1) between neuroticism and both anxiety-depression symptoms and fatigue. Since these effects involve health outcomes (i.e., mental and physical symptoms), differences in stress exposure within twin pairs may partly explain these findings. Several studies have shown that higher levels of neuroticism are associated with greater exposure and reactivity to stressors [63, 64]. In addition, people higher in neuroticism typically exhibit less adequate coping strategies such as self-blame or denial [65]. Furthermore, neuroticism-related stress hyper-reactivity appears to cause an increased sensitization to pain and stress [66], making the more stress-exposed twin in a pair gradually more different from his or her co-twin.

A second common genetic factor, independent of neuroticism and specific to anxiety-depression and both MS and fatigue symptoms, was also identified. This finding is consistent with previous twin research on personality and psychopathology, showing that there is substantial, but not complete, overlap between the genetic factors that influence individual variation in neuroticism and those that increase liability to anxiety-depression and other internalizing disorders [11]. In the present context, however, the most challenging finding is the detection of independent phenotypic and genetic associations of anxiety-depression symptoms with MS pain and fatigue. As noted, a similar finding of an independent (of neuroticism) genetic association between anxiety-depression and somatic distress has been reported by Hansell et al. [53] in a study of adolescent twins. A first possible explanation is that neuroticism and anxiety-depression may be associated with fatigue via both common and differential effects of allostatic activity. However, in the Maloney et al. study [57] cited above, the association between fatigue and allostatic load was independent of depression scores, although there was a weak but significant correlation between the latter two measures. No measure of neuroticism was included in the study, however. Furthermore, the longitudinal study by Stephan et al. [58] (which showed that neuroticism was linked to allostatic load only at baseline), did not comprise measures of negative affect symptoms, pain, fatigue or other subjective states that conceivably could be related to allostatic load over time. Interestingly, a population-based study [67] showed that high neuroticism and low conscientiousness were both associated with higher levels of the cytokine interleukin-6 (IL-6), which is a commonly used as marker of allostatic load [55]. However, the associations were weak (i.e., correlations in the 0.04–0.07 range), and some studies have failed to find an association between neuroticism and IL-6 [68]. Thus, while immunologic and inflammatory processes are obviously of crucial significance in numerous somatic conditions (including conditions often causing fatigue symptoms), their role as underlying mechanisms in the relationship between neuroticism, anxiety-depression, MS pain, and fatigue symptoms must be regarded as unsettled.

Another possibility is that the relationship between neuroticism, anxiety-depression, pain and fatigue is primarily due to individual differences in symptom awareness and symptom amplification. According to this symptom perception hypothesis [26], people higher in neuroticism focus more on internal states, becoming preoccupied with symptoms and exaggerating their effects (see also [8]). Thus, neuroticism, as well as briefer negative affect states, will primarily influence the experience and reporting of subjective health condition, not objectively verifiable physical symptoms and processes such as elevated blood pressure or inflammatory activity. However, individuals with higher neuroticism levels are vulnerable to a broad range of health problems [23], and several large-scale longitudinal studies have documented that initial neuroticism levels are predictive of somatic and psychiatric morbidity assessed several decades later, including moderate to severe conditions that have lasted for significant periods of time [8, 22]. Furthermore, the simultaneous inclusion of neuroticism and anxiety-depression scores in the regression and Cholesky models in the present study shows that if the association of anxiety-depression with MS pain and fatigue is due to a symptom perception/over-reporting effect, this effect is different from the one caused by individual differences in neuroticism, both phenotypically and genetically.

Although the relationship between pain and fatigue has been the focus of substantial epidemiological research in the past, there are very few studies using genetically informative designs. The strong phenotypic correlation (i.e., r = 0.60) between fatigue and MS pain which emerged in the present study, concur with common clinical observations and previous epidemiological research indicating that pain is a frequent symptom in patients with CFS or CFS-like illness. The diagnostic criteria for CFS contain five painful symptoms (including MS pain and headache), and MS pain is the most common of these, affecting as many as 93% of patients [69]. Furthermore, the genetic correlations between fatigue on the one hand, and neuroticism, anxiety-depression and MS symptoms on the other, were substantial, with the correlation between fatigue and MS symptoms emerging as particularly strong. This last finding is comparable to the genetic correlation reported by Burri et al. [9] between fatigue symptoms and CWP. As expected, there were also environmental factors influencing MS pain and fatigue (as reflected in e.g., a moderate individual-specific environmental correlation), indicating that consequences of life events such as illnesses and accidents may affect both phenotypes.

A first and intuitively appealing hypothesis is that fatigue may be a consequence of chronic or episodic pain, possibly mediated by factors such as exacerbated sleep problems and psychological distress. Alternatively, pain may be more easily experienced when being fatigued due to enhanced pain sensitization [70, 71]. A third possibility is that fatigue and pain are symptomatologically related due to shared risk factors and mechanisms. This hypothesis, according to which a synchronous association between changes in fatigue and pain can be envisioned, has actually gained some support in a longitudinal study [72].

Taken together, the findings from both phenotypic and biometric studies strongly suggest that fatigue and MS symptoms are related largely due to shared genetic factors, not because one symptom cluster causes the other. Thus, the fatigue symptoms often observed in individuals with MS pain or other pain conditions may actually not be due to general effects of chronic pain, such as disturbed sleep or side-effects of medication, but may be part of one, complex syndrome (see also [9]). This conclusion is also in accordance with the main findings from a comprehensive longitudinal study, noted above [72], among adult patients presenting with fatigue in 147 medical practices across the Netherlands. Although a fairly complex pattern of results emerged from the statistical analyses, a model reflecting simultaneous change in pain and fatigue was more strongly supported than models specifying a temporal (and implicitly causal) relationship between the symptoms.

### Clinical and preventive implications

MS pain and other pain conditions, anxiety-depression symptoms, and fatigue affect a significant portion of the general population [2, 18, 73]. Thus, mental health practitioners may encounter patients with such symptoms, which are frequently co-occurring and forming complex syndromes, on a regular basis. Of particular note in this context is our finding of significant individual-specific environmental influences, both cross-effects and a unique factor, explaining nearly 50% of the total variance in fatigue. Even when taking into account that these environmental factors also include error of measurement (e.g., the fatigue questionnaire used is about 80% reliable), the systematic individual-specific environmental variance accounts for a substantial amount of the total variance in fatigue symptoms. Importantly, the sources of these influences—psychological, social and medical causal and maintaining factors—would appear to be particularly useful targets for the development of treatment and prevention approaches [9]. One implication of the current research is that the optimal treatment approach for co-occurring fatigue, pain and negative affect symptoms should simultaneously address both physical and psychological symptoms. Interestingly, recent clinical trials have demonstrated that compared with conventional (more passive) forms of rehabilitation, cognitive behavioral therapy (CBT) and graded exercise therapy (GET) were more effective in reducing the frequency of muscle and joint pain in fatigue patients [69]. Another implication is that prevention strategies should be designed to also target the vulnerability for mental and somatic distress or disorders inherent in neuroticism, a vulnerability existing across the whole lifespan, rather than just treating the subsequent manifestations of those conditions [21, 23].

### Limitations

The present study has notable strengths such as using a genetically informative sample of male and female twins who can be considered representative of a large segment of the Norwegian general population. Still, a couple of limitations should also be noted. First, our assessment was based on self-reports, without physical examinations or other objective data. Thus, clinical conditions that may cause fatigue symptoms were not assessed, and the results may not be directly comparable to those based on clinical samples. As noted by several researchers [61], efforts to identify causes, mechanisms or biological markers of CFS or CFS-like conditions have been relatively unsuccessful, and several definitions of fatigue, based mostly on subjective symptoms, have been proposed [2]. A comparative advantage of the current study is that statistical control for general health indices and demographic variables was conducted in the regression analyses. It can furthermore be argued that subjective symptom reporting is significant and valid in its own right as it represents the symptoms with which patients present to their physicians or other therapists, and milder forms of chronic fatigue are much more common than CFS in primary care. Second, the data were obtained in a specific cohort consisting of middle-aged twins, and associations between fatigue symptoms and psychological, social, and medical characteristics may vary with age. However, individual findings from the current study, such as heritability estimates and genetic correlations between fatigue, pain, and other study variables were broadly in agreement with previous research [9, 10, 12], including Hansell et al.’s study [53] of adolescent twins. Additionally, the relationship between neuroticism, negative affect symptoms, and somatic complaints appears highly robust and has been demonstrated across developmental phases, from childhood to old age [74]. Indeed, the present study demonstrated that neuroticism assessed 13–19 years previously were predictive of current fatigue symptoms.

### Conclusions

In conclusion, our results indicate that a substantial part of the variance in fatigue symptoms can be accounted for by a single underlying factor. Extending previous research, the current study examined the role of neuroticism as a general vulnerability factor that may possibly explain the association of fatigue with anxiety-depression and MS symptoms. Cholesky modeling indicated substantial unique genetic and individual-specific environmental influences on each phenotype. Genetic sources accounted for most of the associations among the four phenotypes, but non-negligible environmental cross-effects were also observed. Moreover, a second common genetic factor, independent of neuroticism and specific to the three other phenotypes, as well as a third factor specific to MS symptoms and fatigue, and a fourth factor unique to fatigue, also emerged in the analyses. These results suggest that both unique and pleiotropic effects are involved in the genetic architecture of the phenotypes. Overall, our findings confirms that self-reported fatigue is linked genetically to personality, negative affect symptoms, and pain processes, and is thus both complex (comprising heterogeneous symptoms, occurring across different conditions) and multifactorial (involving several mechanisms and risk factors).

## Supporting information

S1 Fig Path diagram of the second AE Cholesky model (including neuroticism assessed at time 1). (TIF)

## References

[1] Lamers F, Hickie I, Merikangas KR. Prevalence and correlates of prolonged fatigue in a U.S. sample of adolescents. Am J Psychiatry. 2013;170(5):502–10. doi: 10.1176/appi.ajp.2012.12040454 . 23632835 10.1176/appi.ajp.2012.12040454

[2] Ranjith G. Epidemiology of chronic fatigue syndrome. Occup Med (Lond). 2005;55(1):13–9. doi: 10.1093/occmed/kqi012 . 15699086 10.1093/occmed/kqi012

[3] Harvey SB, Wadsworth M, Wessely S, Hotopf M. The relationship between prior psychiatric disorder and chronic fatigue: evidence from a national birth cohort study. Psychol Med. 2008;38(7):933–40. doi: 10.1017/S0033291707001900 . 17976252 10.1017/S0033291707001900 PMC3196526

[4] Prins JB, van der Meer JW, Bleijenberg G. Chronic fatigue syndrome. Lancet. 2006;367(9507):346–55. doi: 10.1016/S0140-6736(06)68073-2 . 16443043 10.1016/S0140-6736(06)68073-2

[5] Evengard B, Jacks A, Pedersen NL, Sullivan PF. The epidemiology of chronic fatigue in the Swedish Twin Registry. Psychol Med. 2005;35(9):1317–26. doi: 10.1017/S0033291705005052 . 16168154 10.1017/S0033291705005052

[6] Wessely S. The epidemiology of chronic fatigue syndrome. Epidemiol Psichiatr Soc. 1998;7(1):10–24. . 9658678 10.1017/s1121189x00007089

[7] Kato K, Sullivan PF, Evengard B, Pedersen NL. Chronic widespread pain and its comorbidities: a population-based study. Arch Intern Med. 2006;166(15):1649–54. doi: 10.1001/archinte.166.15.1649 . 16908799 10.1001/archinte.166.15.1649

[8] Charles ST, Gatz M, Kato K, Pedersen NL. Physical health 25 years later: The predictive ability of neuroticism. Health Psychol. 2008;27(3):369–78. doi: 10.1037/0278-6133.27.3.369 . 18624602 10.1037/0278-6133.27.3.369

[9] Burri A, Ogata S, Livshits G, Williams F. The Association between Chronic Widespread Musculoskeletal Pain, Depression and Fatigue Is Genetically Mediated. Plos One. 2015;10(11). doi: 10.1371/journal.pone.0140289 26599910 10.1371/journal.pone.0140289 PMC4657992

[10] Sullivan PF, Evengard B, Jacks A, Pedersen NL. Twin analyses of chronic fatigue in a Swedish national sample. Psychol Med. 2005;35(9):1327–36. doi: 10.1017/S0033291705005222 . 16168155 10.1017/S0033291705005222

[11] Hettema JM, Neale MC, Myers JM, Prescott CA, Kendler KS. A population-based twin study of the relationship between neuroticism and internalizing disorders. Am J Psychiat. 2006;163(5):857–64. doi: 10.1176/ajp.2006.163.5.857 . 16648327 10.1176/ajp.2006.163.5.857

[12] Hickie I, Kirk K, Martin N. Unique genetic and environmental determinants of prolonged fatigue: a twin study. Psychol Med. 1999;29(2):259–68. . 10218917 10.1017/s0033291798007934

[13] Farmer A, Scourfield J, Martin N, Cardno A, McGuffin P. Is disabling fatigue in childhood influenced by genes? Psychol Med. 1999;29(2):279–82. doi: 10.1017/S0033291798008095 10218919 10.1017/s0033291798008095

[14] Schur E, Afari N, Goldberg J, Buchwald D, Sullivan PF. Twin analyses of fatigue. Twin Res Hum Genet. 2007;10(5):729–33. doi: 10.1375/twin.10.5.729 17903114 10.1375/twin.10.5.729 PMC2953372

[15] Ball HA, Siribaddana SH, Sumathipala A, Kovas Y, Glozier N, Rijsdijk F, et al Genetic and environmental contributions to the overlap between psychological, fatigue and somatic symptoms: a twin study in Sri Lanka. Twin Res Hum Genet. 2011;14(1):53–63. doi: 10.1375/twin.14.1.53 . 21314256 10.1375/twin.14.1.53 PMC3066854

[16] Kato K, Sullivan PF, Evengard B, Pedersen NL. Premorbid predictors of chronic fatigue. Arch Gen Psychiatry. 2006;63(11):1267–72. doi: 10.1001/archpsyc.63.11.1267 . 17088507 10.1001/archpsyc.63.11.1267

[17] Roy-Byrne P, Afari N, Ashton S, Fischer M, Goldberg J, Buchwald D. Chronic fatigue and anxiety/depression: a twin study. Br J Psychiatry. 2002;180:29–34. . 11772848 10.1192/bjp.180.1.29

[18] Kato K, Sullivan PF, Evengard B, Pedersen NL. A population-based twin study of functional somatic syndromes. Psychol Med. 2009;39(3):497–505. doi: 10.1017/S0033291708003784 18578896 10.1017/S0033291708003784 PMC3947533

[19] Hur YM, Burri A, Spector TD. The genetic and environmental structure of the covariation among the symptoms of insomnia, fatigue, and depression in adult females. Twin Res Hum Genet. 2012;15(6):720–6. doi: 10.1017/thg.2012.60 . 22999525 10.1017/thg.2012.60

[20] van der Windt DA, Dunn KM, Spies-Dorgelo MN, Mallen CD, Blankenstein AH, Stalman WA. Impact of physical symptoms on perceived health in the community. J Psychosom Res. 2008;64(3):265–74. doi: 10.1016/j.jpsychores.2007.10.003 . 18291241 10.1016/j.jpsychores.2007.10.003

[21] Lahey BB. Public health significance of neuroticism. Am Psychol. 2009;64(4):241–56. doi: 10.1037/a0015309 . 19449983 10.1037/a0015309 PMC2792076

[22] Neeleman J, Sytema S, Wadsworth M. Propensity to psychiatric and somatic ill-health: evidence from a birth cohort. Psychol Med. 2002;32(5):793–803. doi: 10.1017/S0033291702005901 12171374 10.1017/s0033291702005901

[23] Cuijpers P, Smit F, Penninx BW, de Graaf R, ten Have M, Beekman AT. Economic costs of neuroticism: a population-based study. Arch Gen Psychiat. 2010;67(10):1086–93. doi: 10.1001/archgenpsychiatry.2010.130 . 20921124 10.1001/archgenpsychiatry.2010.130

[24] Berthelsen M, Pallesen S, Bjorvatn B, Knardahl S. Shift schedules, work factors, and mental health among onshore and offshore workers in the Norwegian petroleum industry. Ind Health. 2015;53(3):280–92. doi: 10.2486/indhealth.2014-0186 25740007 10.2486/indhealth.2014-0186 PMC4466879

[25] Goubert L, Crombez G, Van Damme S. The role of neuroticism, pain catastrophizing and pain-related fear in vigilance to pain: a structural equations approach. Pain. 2004;107(3):234–41. . 14736586 10.1016/j.pain.2003.11.005

[26] Watson D, Pennebaker JW. Health complaints, stress, and distress—exploring the central role of negative affectivity. Psychol Rev. 1989;96(2):234–54. doi: 10.1037/0033-295x.96.2.234 2710874 10.1037/0033-295x.96.2.234

[27] De Vries J, Van Heck GL. Fatigue: relationships with basic personality and temperament dimensions. Pers Indiv Differ. 2002;33(8):1311–24. doi: 10.1016/S0191-8869(02)00015-6

[28] Poeschla B, Strachan E, Dansie E, Buchwald DS, Afari N. Chronic fatigue and personality: a twin study of causal pathways and shared liabilities. Ann Behav Med. 2013;45(3):289–98. doi: 10.1007/s12160-012-9463-5 . 23361410 10.1007/s12160-012-9463-5 PMC3643988

[29] Nilsen TS, Knudsen GP, Gervin K, Brandt I, Roysamb E, Tambs K, et al The Norwegian Twin Registry from a public health perspective: a research update. Twin Res Hum Genet. 2013;16(1):285–95. doi: 10.1017/thg.2012.117 . 23186607 10.1017/thg.2012.117

[30] Magnus P, Berg K, Nance WE. Predicting zygosity in Norwegian twin pairs born 1915–1960. Clin Genet. 1983;24(2):103–12. 6577993 10.1111/j.1399-0004.1983.tb02220.x

[31] Eysenck SBG, Eysenck HJ, Barrett P. A Revised Version of the Psychoticism Scale. Pers Indiv Differ. 1985;6(1):21–9. doi: 10.1016/0191-8869(85)90026-1

[32] Birley AJ, Gillespie NA, Heath AC, Sullivan PF, Boomsma DI, Martin NG. Heritability and nineteen-year stability of long and short EPQ-R neuroticism scales. Pers Indiv Differ. 2006;40(4):737–47. doi: 10.1016/j.paid.2005.09.005

[33] Tambs K, Harris JR, Magnus P. Sex-specific causal factors and effects of common environment for symptoms of anxiety and depression in twins. Behav Genet. 1995;25(1):33–44. doi: 10.1007/BF02197240 . 7755517 10.1007/BF02197240

[34] Costa PT, McCrae RR. Revised NEO Personality Inventory (NEO-PI-R) and NEO Five-Factor Inventory (NEO-FFI). Odessa, FL: Psychological Assessment Resources; 1992.

[35] Martinsen Ø, Nordvik H, Østbø LE. NEO-PI-R. Oslo: Gyldendal; 2003.

[36] Roberts BW, DelVecchio WF. The rank-order consistency of personality traits from childhood to old age: a quantitative review of longitudinal studies. Psychol Bull. 2000;126(1):3–25. . 10668348 10.1037/0033-2909.126.1.3

[37] Ferguson CJ. A Meta-Analysis of Normal and Disordered Personality Across the Life Span. J Pers Soc Psychol. 2010;98(4):659–67. doi: 10.1037/a0018770 20307136 10.1037/a0018770

[38] Brähler E, Scheer J. Der Giessener Beschwerdebogen Handbuch [Manual of the Giessen Symptom Checklist]. Bern: Hans Huber; 1983.

[39] Vassend O, Lian L, Andersen HT. Norwegian versions of the NEO-Personality Inventory, Symptom Checklist 90 Revised, and Giessen Subjective Complaints List: I. Tidsskrift for Norsk Psykologforening [Journal of the Norwegian Psychological Association]. 1992;29(12):1150–60.

[40] Vassend O, Skrondal A. The Role of Negative Affectivity in Self assessment of Health: A Structural Equation Approach. J Health Psychol. 1999;4(4):465–82. doi: 10.1177/135910539900400402 . 22021640 10.1177/135910539900400402

[41] Howren MB, Suls J. The symptom perception hypothesis revised: Depression and anxiety play different roles in concurrent and retrospective physical symptom reporting. J Pers Soc Psychol. 2011;.100(1):pp. doi: 10.1037/a0021715 . 21219079 10.1037/a0021715

[42] Derogatis LR, Lipman RS, Rickels K, Uhlenhuth EH, Covi L. The Hopkins Symptom Checklist (HSCL): A self-report symptom inventory. Behav Sci. 1974;19(1):1–15. doi: 10.1002/bs.3830190102 . 4808738 10.1002/bs.3830190102

[43] Feightner JW, Worrall G. Early detection of depression by primary care physicians. CMAJ. 1990;142(11):1215–20. . 2188720 PMC1452592

[44] Reichborn-Kjennerud T, Stoltenberg C, Tambs K, Roysamb E, Kringlen E, Torgersen S, et al Back-neck pain and symptoms of anxiety and depression: a population-based twin study. Psychol Med. 2002;32(6):1009–20. . 12214782 10.1017/s0033291702005950

[45] Gjerde LC, Roysamb E, Czajkowski N, Reichborn-Kjennerud T, Orstavik RE, Kendler KS, et al Strong genetic correlation between interview-assessed internalizing disorders and a brief self-report symptom scale. Twin Res Hum Genet. 2011;14(1):64–72. doi: 10.1375/twin.14.1.64 . 21314257 10.1375/twin.14.1.64 PMC3081885

[46] Moum T. Self-Assessed Health among Norwegian Adults. Soc Sci Med. 1992;35(7):935–47. doi: 10.1016/0277-9536(92)90108-3 1411694 10.1016/0277-9536(92)90108-3

[47] Carlin JB, Gurrin LC, Sterne JAC, Morley R, Dwyer T. Regression models for twin studies: a critical review. Int J Epidemiol. 2005;34(5):1089–99. doi: 10.1093/ije/dyi153 16087687 10.1093/ije/dyi153

[48] Neale MC, Roysamb E, Jacobson K. Multivariate genetic analysis of sex limitation and G x E interaction. Twin Res Hum Genet. 2006;9(4):481–9. doi: 10.1375/183242706778024937 . 16899154 10.1375/183242706778024937 PMC4246510

[49] Carey G. Cholesky problems. Behav Genet. 2005;35(5):653–65. doi: 10.1007/s10519-005-5355-9 16184491 10.1007/s10519-005-5355-9

[50] Boker S, Neale M, Maes H, Wilde M, Spiegel M, Brick T, et al OpenMx: An open source extended structural equation modeling framework. Psychometrika. 2011;76(2):306–17. doi: 10.1007/s11336-010-9200-6 23258944 10.1007/s11336-010-9200-6 PMC3525063

[51] Akaike H. Factor analysis and AIC. Psychometrika. 1987;52(3):317–32. doi: 10.1007/Bf02294359

[52] Mogil JS. Sex differences in pain and pain inhibition: multiple explanations of a controversial phenomenon. Nat Rev Neurosci. 2012;13(12):859–66. doi: 10.1038/nrn3360 . 23165262 10.1038/nrn3360

[53] Hansell NK, Wright MJ, Medland SE, Davenport TA, Wray NR, Martin NG, et al Genetic co-morbidity between neuroticism, anxiety/depression and somatic distress in a population sample of adolescent and young adult twins. Psychol Med. 2012;42(6):1249–60. doi: 10.1017/S0033291711002431 . 22051348 10.1017/S0033291711002431

[54] McEwen BS, Stellar E. Stress and the individual. Mechanisms leading to disease. Arch Intern Med. 1993;153(18):2093–101. . 8379800

[55] Juster RP, McEwen BS, Lupien SJ. Allostatic load biomarkers of chronic stress and impact on health and cognition. Neurosci Biobehav R. 2010;35(1):2–16. doi: 10.1016/j.neubiorev.2009.10.002 19822172 10.1016/j.neubiorev.2009.10.002

[56] Edes AN, Crews DE. Allostatic load and biological anthropology. Am J Phys Anthropol. 2017;162 Suppl 63:44–70. doi: 10.1002/ajpa.23146 . 28105719 10.1002/ajpa.23146

[57] Maloney EM, Boneva R, Nater UM, Reeves WC. Chronic fatigue syndrome and high allostatic load: results from a population-based case-control study in Georgia. Psychosom Med. 2009;71(5):549–56. doi: 10.1097/PSY.0b013e3181a4fea8 . 19414615 10.1097/PSY.0b013e3181a4fea8

[58] Stephan Y, Sutin AR, Luchetti M, Terracciano A. Allostatic Load and Personality: A 4-Year Longitudinal Study. Psychosom Med. 2016;78(3):302–10. doi: 10.1097/PSY.0000000000000281 . 26716813 10.1097/PSY.0000000000000281 PMC5481782

[59] Deary V, Hagenaars SP, Harris SE, Hill WD, Davies G, Liewald DC, et al Genetic contributions to self-reported tiredness. Mol Psychiatry. 2017 doi: 10.1038/mp.2017.5 . 28194004 10.1038/mp.2017.5 PMC5822465

[60] Gale CR, Hagenaars SP, Davies G, Hill WD, Liewald DCM, Cullen B, et al Pleiotropy between neuroticism and physical and mental health: findings from 108038 men and women in UK Biobank. Transl Psychiat. 2016;6, e791; doi: 10.1038/tp.2016.56 27115122 10.1038/tp.2016.56 PMC4872414

[61] Landmark-Hoyvik H, Reinertsen KV, Loge JH, Kristensen VN, Dumeaux V, Fossa SD, et al The Genetics and Epigenetics of Fatigue. Pm&R. 2010;2(5):456–65. doi: 10.1016/j.pmrj.2010.04.003 20656628 10.1016/j.pmrj.2010.04.003

[62] Wang T, Yin J, Miller AH, Xiao C. A systematic review of the association between fatigue and genetic polymorphisms. Brain Behav Immun. 2017 doi: 10.1016/j.bbi.2017.01.007 . 28089639 10.1016/j.bbi.2017.01.007 PMC5947855

[63] Mroczek DK, Almeida DM. The effect of daily stress, personality, and age on daily negative affect. J Pers. 2004;72(2):355–78. doi: 10.1111/j.0022-3506.2004.00265.x 15016068 10.1111/j.0022-3506.2004.00265.x

[64] Bolger N, Schilling EA. Personality and the Problems of Everyday Life—the Role of Neuroticism in Exposure and Reactivity to Daily Stressors. J Pers. 1991;59(3):355–86. doi: 10.1111/j.1467-6494.1991.tb00253.x 1960637 10.1111/j.1467-6494.1991.tb00253.x

[65] Carver CS, Connor-Smith J. Personality and Coping. Annu Rev Psychol. 2010;61:679–704. doi: 10.1146/annurev.psych.093008.100352 19572784 10.1146/annurev.psych.093008.100352

[66] Wilhelmsen I. Biological sensitisation and psychological amplification: Gateways to subjective health complaints and somatoform disorders. Psychoneuroendocrino. 2005;30(10):990–5. doi: 10.1016/j.psyneuen.2005.01.011 15963653 10.1016/j.psyneuen.2005.01.011

[67] Sutin AR, Terracciano A, Deiana B, Naitza S, Ferrucci L, Uda M, et al High Neuroticism and low Conscientiousness are associated with interleukin-6. Psychol Med. 2010;40(9):1485–93. doi: 10.1017/S0033291709992029 19995479 10.1017/S0033291709992029 PMC2933046

[68] Chapman BP, Khan A, Harper M, Stockman D, Fiscella K, Walton J, et al Gender, race/ethnicity, personality, and interleukin-6 in urban primary care patients. Brain Behav Immun. 2009;23(5):636–42. doi: 10.1016/j.bbi.2008.12.009 19162168 10.1016/j.bbi.2008.12.009 PMC2694851

[69] Bourke JH, Johnson AL, Sharpe M, Chalder T, White PD. Pain in chronic fatigue syndrome: response to rehabilitative treatments in the PACE trial. Psychol Med. 2014;44(7):1545–52. doi: 10.1017/S0033291713002201 . 23967878 10.1017/S0033291713002201

[70] Meeus M, Nijs J. Central sensitization: a biopsychosocial explanation for chronic widespread pain in patients with fibromyalgia and chronic fatigue syndrome. Clin Rheumatol. 2007;26(4):465–73. doi: 10.1007/s10067-006-0433-9 . 17115100 10.1007/s10067-006-0433-9 PMC1820749

[71] Nijs J, Van de Velde B, De Meirleir K. Pain in patients with chronic fatigue syndrome: does nitric oxide trigger central sensitisation? Med Hypotheses. 2005;64(3):558–62. doi: 10.1016/j.mehy.2004.07.037 . 15617866 10.1016/j.mehy.2004.07.037

[72] Nijrolder I, van der Windt DA, Twisk JW, van der Horst HE. Fatigue in primary care: longitudinal associations with pain. Pain. 2010;150(2):351–7. doi: 10.1016/j.pain.2010.05.030 . 20573449 10.1016/j.pain.2010.05.030

[73] Bergman S, Herrstrom P, Hogstrom K, Petersson IF, Svensson B, Jacobsson LT. Chronic musculoskeletal pain, prevalence rates, and sociodemographic associations in a Swedish population study. J Rheumatol. 2001;28(6):1369–77. . 11409133

[74] Friedman HS, Kern ML. Personality, well-being, and health. Annu Rev Psychol. 2014:719–42. doi: 10.1146/annurev-psych-010213-115123 . 24405364 10.1146/annurev-psych-010213-115123

