# Supplementary figures and images for: Fatigue symptoms in relation to neuroticism, anxiety-depression, and musculoskeletal pain. A longitudinal twin study
Source: PLoS One. 2018 Jun 7;13(6):e0198594. doi: 10.1371/journal.pone.0198594 (PMC5991664; doi:10.1371/journal.pone.0198594)

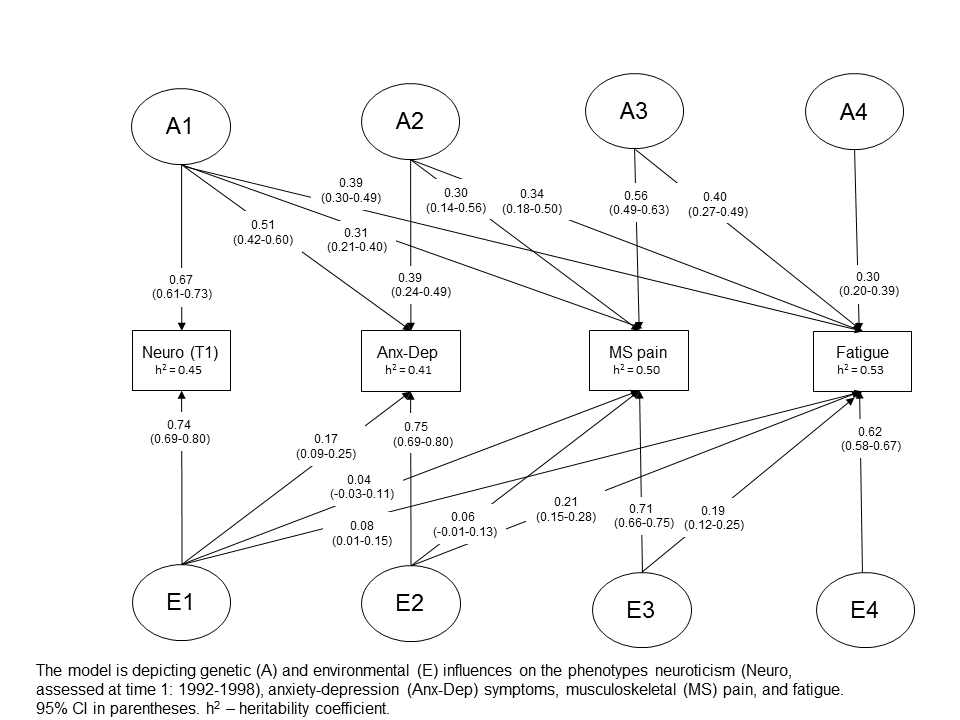

Supplement: S1 Fig — (TIF) [file pone.0198594.s001.tif]
